# *Carrot yellow leaf virus* Is Associated with Carrot Internal Necrosis

**DOI:** 10.1371/journal.pone.0109125

**Published:** 2014-11-03

**Authors:** Ian P. Adams, Anna Skelton, Roy Macarthur, Tobias Hodges, Howard Hinds, Laura Flint, Palash Deb Nath, Neil Boonham, Adrian Fox

**Affiliations:** 1 Centre for Crop Protection, Food and Environment Research Agency, Sand Hutton, York, United Kingdom; 2 RootCrop Ltd., Hoveringham, Nottinghamshire, United Kingdom; 3 University of York, York, United Kingdom; 4 Department of Plant Pathology, Assam Agricultural University, Jorhat, India; Nanjing Agricultural University, China

## Abstract

Internal necrosis of carrot has been observed in UK carrots for at least 10 years, and has been anecdotally linked to virus infection. In the 2009 growing season some growers had up to 10% of yield with these symptoms. Traditional diagnostic methods are targeted towards specific pathogens. By using a metagenomic approach with high throughput sequencing technology, other, as yet unidentified causes of root necrosis were investigated. Additionally a statistical analysis has shown which viruses are most closely associated with disease symptoms. Carrot samples were collected from a crop exhibiting root necrosis (102 Affected: 99 Unaffected) and tested for the presence of the established carrot viruses: *Carrot red leaf virus (CtRLV), Carrot mottle virus (CMoV)*, Carrot red leaf associated viral RNA (CtRLVaRNA) and *Parsnip yellow fleck virus (PYFV)*. The presence of these viruses was not associated with symptomatic carrot roots either as single viruses or in combinations. A sub-sample of carrots of mixed symptom status was subjected to MiSeq sequencing. The results from these tests suggested *Carrot yellow leaf virus* (CYLV) was associated with symptomatic roots. Additionally a novel Torradovirus, a novel Closterovirus and two novel Betaflexiviradae related plant viruses were detected. A specific diagnostic test was designed for CYLV. Of the 102 affected carrots, 98% were positive for CYLV compared to 22% of the unaffected carrots. From these data we conclude that although we have yet to practically demonstrate a causal link, CYLV appears to be strongly associated with the presence of necrosis of carrots.

## Introduction

For at least 10 years UK growers have reported carrot roots exhibiting internal necrosis around the root core extending from crown to tip, and these have been anecdotally associated with the presence of viruses. The 2009 growing season saw some growers with up to 10% of yield affected by these symptoms, although symptom development appeared to be locally significant, with many growers reporting no evidence of root symptoms in crops. It is difficult to grade out affected carrots because the symptoms tend to be internal. Results of a limited survey in 2010 [1] suggested a possible association between the presence of root necrosis symptoms and virus infection. However, a large proportion of the carrots tested in this earlier study were negative when tested for PYFV or the Carrot Motley Dwarf complex (CMD) of viruses. This finding raised the question of other viruses being a cause of the development of carrot root necrosis.

Globally more than 30 viruses are known to affect carrot [2]. The principal viruses known to affect commercial carrot crops in the UK are *Parsnip yellow fleck virus* (PYFV) and the CMD Complex consisting of *Carrot red leaf virus* (CtRLV), *Carrot mottle virus* (CMoV) and Carrot red leaf associated RNA (CtRLVaRNA). The importance of PYFV and CtRLV as viruses causing economic damage have been recognised for over 20 years due to the foliar symptoms (CtRLV) and viral die-back of seedlings (PYFV) [3]. In the UK these viruses affect carrot crops only sporadically but when they do occur they can be devastating. Other carrot viruses are known to occur in the UK, however, their effects are not clear.

*Carrot yellow leaf virus* (CYLV) (Genus *Closterovirus*, Family *Closteroviridae*) was first isolated from carrot samples showing yellowing foliage from Japan [4] and described on the basis of particle morphology; measurement by Electron Microscopy (1,600×12 nm, 3.7 nm Helical pitch); being limited to phloem and having characteristics of closterovirus infection. Bem and Murant [5] later described a series of viruses found in the UK from hogweed (*Heracleum sphondylium*), among which were the filamentous viruses Hogweed 2 virus with a particle size of 700–750 nm, and Hogweed 6 virus (HV6) with a particle size of 1400 nm, and being transmissible by aphids these were tentatively assigned to the genus Closterovirus [5]. Hogweed 2 virus, with shorter particles, was characterised as *Heracleum latent virus* (HLV) [6]. Hogweed 6 virus was subsequently shown to be transmitted by aphids including *Cavariella* spp. and to act as the helper virus for the transmission of HLV [7]. Murant again reported the length of HV6 as 1400 nm and that mechanical inoculation of HV6 was unsuccessful [8]. During a subsequent survey of umbellifers in the Netherlands [3] the virus previously reported by Murant [6], [7], [8] as HV6 was considered to be CYLV on the basis of host range similarity and the ability to facilitate co-transmission of HLV.

A virus isolated from carrot in the Netherlands was reported as ‘resembling CYLV’ due to host symptoms; the presence of closterovirus-like particles; and a host range that was different to *Beet yellows virus* [9]. Murant reported that sap inoculation of CYLV had been unsuccessful [8], van Dijk and Bos [9] reported poor sap transmissibility into *Nicotiana benthamiana*, but subsequent attempts to mechanically transmit this virus back into carrot were unsuccessful.

An isolate of an unknown closterovirus from a German carrot sample exhibiting foliar yellowing symptoms was shown to be CYLV through molecular characterisation [10]. There is no literature linking any carrot viruses with necrotic root symptoms.

Detection of carrot viruses is currently carried out using conventional PCR methods, which give efficient, specific detection of single targets. With the use of degenerate primer sets they can be used to detect a number of pathogens of the same genus [10]. However, such targeted testing will not reveal the presence of unexpected or unknown viruses. Even multi-target approaches such as micro-array based methods [11] are unlikely to reveal the presence of complete unknowns, unless cross-hybridisation to known close relatives occurs. A more efficient approach would be to use a ‘non-targeted’ method such as next generation (high throughput) sequencing for diagnosis of viral pathogens. These techniques have been successfully deployed in plant pathology for the detection of novel viruses [12], [13], [14] or for the diagnosis of unusual strains of plant viruses [15]. Such approaches are rapidly becoming more cost effective as the high throughput platforms develop. Previous reports utilising this technology have tended to identify the presence of a novel or unusual virus in single or pooled samples and then use the sequence generated to design targeted diagnostics to validate the finding from the original sample. Putting these findings into a broader context of field pathology is more challenging.

To definitively link a pathogenic cause to an observed symptom it is necessary to demonstrate Koch's postulates, the isolation from a diseased individual of a pure culture of a pathogen which is then used to induce symptoms in a previously healthy host. These requirements, first described in 1890 [16], were intended to set a standard methodology for proof of a causal relationship. As viruses are obligate pathogens, it is not possible to obtain a ‘pure culture’, in addition some viruses can be difficult to transmit and the specific transmission mechanism of a new virus may not be known. For diseases induced by a single virus species Koch's postulates may be satisfied in their broadest interpretation i.e. a pathogen is isolated from a symptomatic plant into an experimental host and then back-inoculated into the original host species to try and replicate the original symptom. Attempts have also been made to look at causation in light of developments in molecular detection [16]. However, where a complex of viruses may be affecting a host or where there may be environmental or agronomic influences on symptom development (temperature, moisture, time from exposure, time in ground/crop growth stage, etc) trying to link detection of pathogen/s with a symptom using a conventional cause-and-effect relation is often not possible. Therefore statistical approaches have been employed to demonstrate the possible influence of single or multiple pathogens on the expression of symptoms within a sampled population [17].

This paper describes a study of the potential causes of carrot internal necrosis using RT_PCR of common carrot viruses and next generation sequencing in carrots with and without symptoms of necrosis and a statistical approach to associate particular viruses with the incidence of necrotic symptoms.

## Materials and Methods

### Ethics statement

The carrots were obtained with the permission of Rodger Hobson, Hobson's Farming and no further permissions were required. The samples were taken post-harvest so there was no damage to endangered or protected species.

### Source of carrot samples

The crop of carrots sampled were grown at Gothic Back Field, York, UK (OS Grid Ref: SE 65486 445297; Latitude 53.899711, Longitude -1.0048628). On the grading line carrots were cut and examined for symptoms, of these 3% of the 3300 individual carrots examined contained necrotic symptoms. Some of these carrots had surface necrosis which could have been graded out, others had only internal necrosis, and there were also carrots sampled with a combination of internal and external symptoms. From these samples 102 carrot roots were selected which were affected by disease (i.e. necrotic/symptomatic) and 99 carrot roots were selected which were un-affected (non-necrotic/asymptomatic). As the carrots were sampled on the grading line no assessment of foliar symptoms could be made.

### Sap inoculations

Five plants of each of the standard indicators *Nicotiana benthamiana, N. debneyi, N. hesperis, N. tobaccum* (cv White Burley), *N. occidentalils* (P1), *Chenopodium quinoa*, and *C. amaranticolor*, Tomato and the umbelliferous plants Carrot, Chervil and Coriander were inoculated from carrot samples using the methods described in Hill [18]. Control and non-inoculated plants were maintained for all species. Plants were maintained in a green house with a mean temperature of 22°C with an 18 hr. photoperiod and assessed for symptoms weekly.

### Reverse Transcription PCR

RNA was extracted from carrot roots by magnetic bead extraction using Invimag Virus DNA/RNA mini-kit (Invitek GMBH). Conventional RT-PCR was carried out for the presence of the four carrot viruses known to be common in the UK namely *Parsnip yellow fleck virus* [19] and the viruses of the Carrot Motley Dwarf complex, *Carrot red leaf virus, Carrot Mottle virus* [*20*] and Carrot red leaf associated viral RNA [21]. All RT-PCR reactions were carried out using Verso 1-Step RT-PCR ReddyMix Kit (Thermo Scientific) on a GeneAmp 9700 (Applied Biosystems) (Annealing Temperature 50°C).

### High Throughput Sequencing

RNA was extracted from 12 affected and 12 unaffected carrots using an RNeasy kit (Qiagen, UK). TruSeq RNA Indexed sequencing libraries (Illumina) were then prepared following the manufacturers recommended protocols, before being sequenced on 2 500 cycle v2 flow cells using MiSeq Sequencer (Illumina). The resulting sequences were trimmed to remove low-quality nucleotides from the 3′ end, using a Phred score threshold of 30 and the *bwa* trimming approach implemented in SolexaQA [22], and assembled using Trinity [23]. Contigs produced were then compared to the GenBank protein database using BLASTx + [24] and viral reads extracted using MEGAN [25], [26]. Open reading frames were identified using Vecor NTi v11 (Invitrogen, UK) and alignments and phylogenetic trees produced using Mega5 with 500 bootstrapped replicates [27]. To determine the number of viral reads in the affected and unaffected samples reads were mapped back to the genomes of identified viruses using bwa *aln sampe* [28] and the numbers of matched read pairs extracted using Samtools [29]. To normalize mapped read counts against the length of genome and total number of reads, the values are reported as mapped reads per kilobase of viral genome per million reads (RPKM), an approach originally introduced for the comparison of mRNA abundance in differential expression analyses [30]. The fastq data produced during the project were submitted to the short read achive acc: SRP042501.

### Real-time PCR

Real-time (TaqMan) primers and probes were designed using Primer Express 2 with sequences from GenBank and derived from the sequencing in this study when available (Applied Biosystems). Real-time RT-PCR was performed on previously extracted RNA in 96 well plates on an ABI 7900 instrument (Applied Biosystems). Reactions consisted of 1× buffer A (Applied Biosystems), 0.2 mM of each dNTP, 5.5 mM MgCl_2_, 0.025 U/µl AmpliTaq Gold (Applied Biosystems), 0.4 U/µl Revertaid (Fermentas), 300 nM of each primer, 100 nM of probe and 1 µl of extracted RNA (concentration as extracted) to give a final reaction volume of 25 µl. The cycling conditions used were: 30 min at 48°C, 10 min at 95°C, then 40 times, 15 sec at 95°C and 1 min at 60°C. Negative controls consisted of water replacing the template. Results were scored as positive or negative for CYLV based on presence or absence of amplification after 40 cycles.

### Statistical analysis

The extent to which necrosis may be caused by viruses was assessed by counting the proportion of carrots that are necrotic and testing equal numbers of necrotic and non-necrotic carrots for the presence of viruses. Hence we observed three proportions:


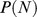
: the proportion of carrots that were necrotic.


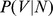
: the proportion of necrotic carrots that contained a virus,


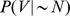
: the proportion of non-necrotic carrots that contained a virus.

Additionally estimates of three proportions were derived:


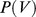
: the proportion of carrots that contained virus,


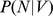
: the proportion of carrots with a virus that are necrotic


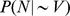
: the proportion of carrots without a virus that are necrotic.

Estimates were derived from the law of total probability



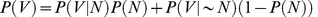
(Equation 1)


and Bayes' Theorem



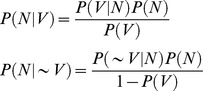
(Equation 2)


The size of the uncertainty associated with observed proportions was estimated using a Modified Jeffreys interval [1], where given x ‘positives’ out on n observations the probability p underlying the observed proportion is with confidence 1-α



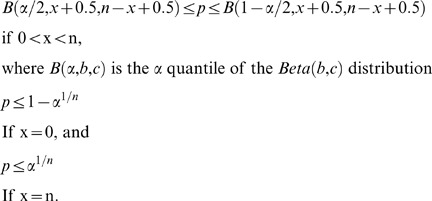
(Equation 3)


The size of the predicted effect of removing a virus, on the prevalence of necrosis expressed as the proportional reduction in prevalence was estimated using



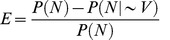
(Equation 4)


The uncertainty associated with derived estimates was estimated by generating independent random (uniform (0,1)) quantiles for each of the observed proportions (Equation 3) and calculating derived values using Equations 1 and 2. 95% confidence intervals were taken from the 2.5^th^ and 97.5^th^ percentiles of 10000 derived values.

## Results

### Sampling

**Figure 1:**
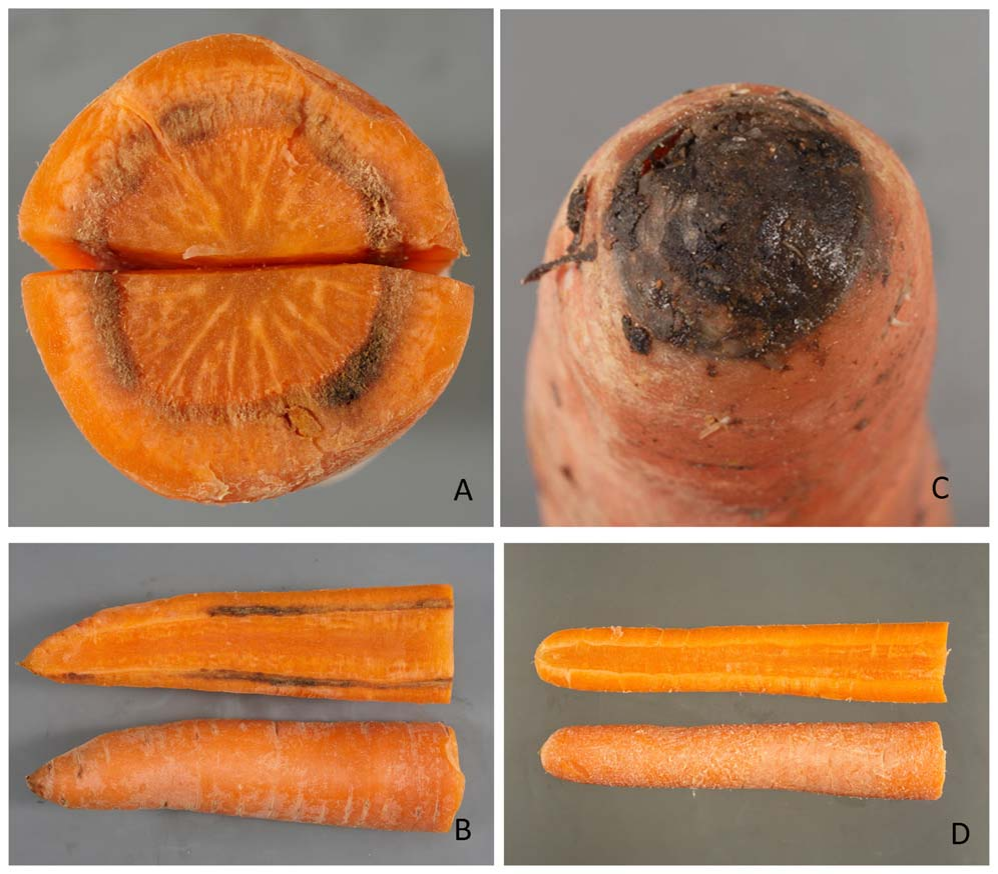
Examples of symptoms of ‘affected’ carrot samples (a) Cross section of carrot root showing internal necrosis around the root core. (b) Internal necrosis of carrot root along the root core. *(c)* External necrosis of the root tip. (d) Unaffected carrot.

A sample of 3300 carrots were examined for necrotic root symptoms. The prevalence of symptoms was estimated from this sample to be approximately 3%. The 102 necrotic/symptomatic carrot samples found within the sample were taken for analysis. These roots exhibited a range of symptoms which included cases of internal and external necrosis. (See Figure 1). 99 asymptomatic/non-necrotic carrots were also taken for analysis.

### Reverse-Transcriptase PCR Testing

**Figure 2:**
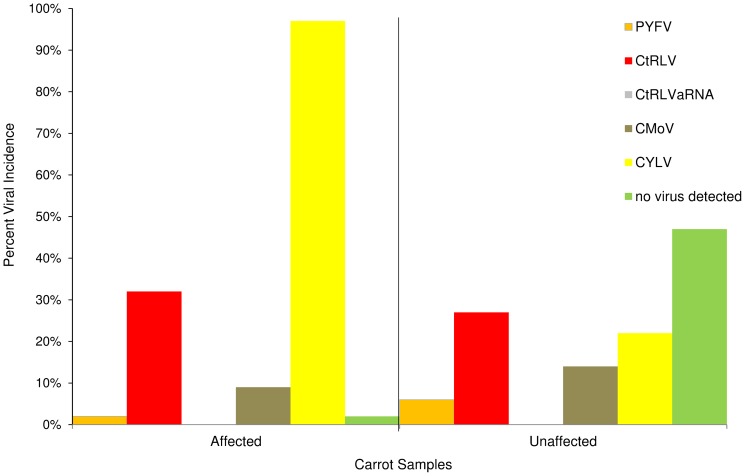
Percent virus incidence in carrot roots from the total field sample of 102 affected and 99 unaffected carrots for the presence of viruses including CYLV, presented as a percentage of carrots with necrosis symptoms (affected) and without necrosis symptoms (unaffected) where virus was detected by PCR or TaqMan.

The sample of affected (symptomatic) and unaffected carrots were initially tested using conventional RT-PCR assays for the presence of the carrot viruses which are known to be common in the UK (PYFV, CMoV, CtRLV, CtRLVaRNA). Results from this testing are presented in Figure 2 which shows the percent of affected or unaffected carrots which contained the viruses. Approximately equal proportions of affected and unaffected carrots were positive for PYFV and CMD viruses (37% affected, 38% unaffected). The two groups also contained similar incidences of CtRLV (33% affected, 27% unaffected) and CMoV (9% affected, 14% unaffected). No CtRLVaRNA was detected from carrots of either symptom status. As RT-PCR results from the two groupings were broadly comparable no influence of the viruses upon the incidence of necrosis symptoms was detected. A subsample of carrots was subsequently tested using high throughput sequencing to investigate the presence of non-target pathogens.

### High Throughput Sequencing

**Figure 3:**
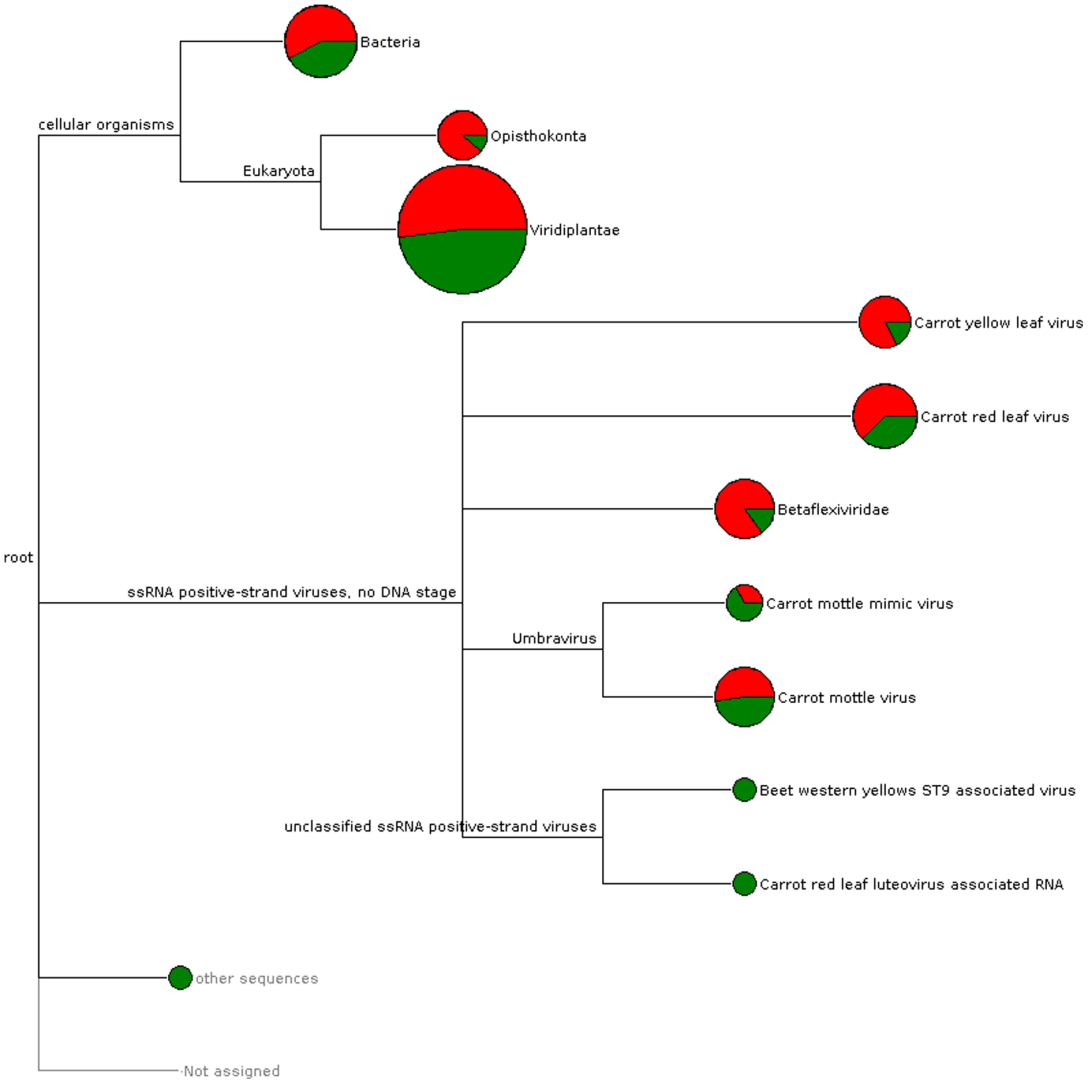
MEGAN derived Phylogram showing the putative identification of contigs in the Sequencing data. Circle size is derived from the number of contigs assigned to each taxa Red: affected, Green: unaffected.

Indexed pair end reads (16225604 in total) with a sequence length of 2×250 bp were obtained from 12 affected and 12 unaffected carrot RNA extracts. A phylogram (figure 3), produced in MEGAN [25], [26] details the viruses found in the affected and unaffected samples. *Carrot yellow leaf virus* (CYLV) was by far the most prevalent virus in the affected samples (RPKM  = 221.3) but it was not common in the unaffected samples (RPKM  = 2.2). Comparison of the MEGAN pylograms for affected and unaffected carrots did not reveal any fungi or bacteria unique to the affected carrots.

**Figure 4:**
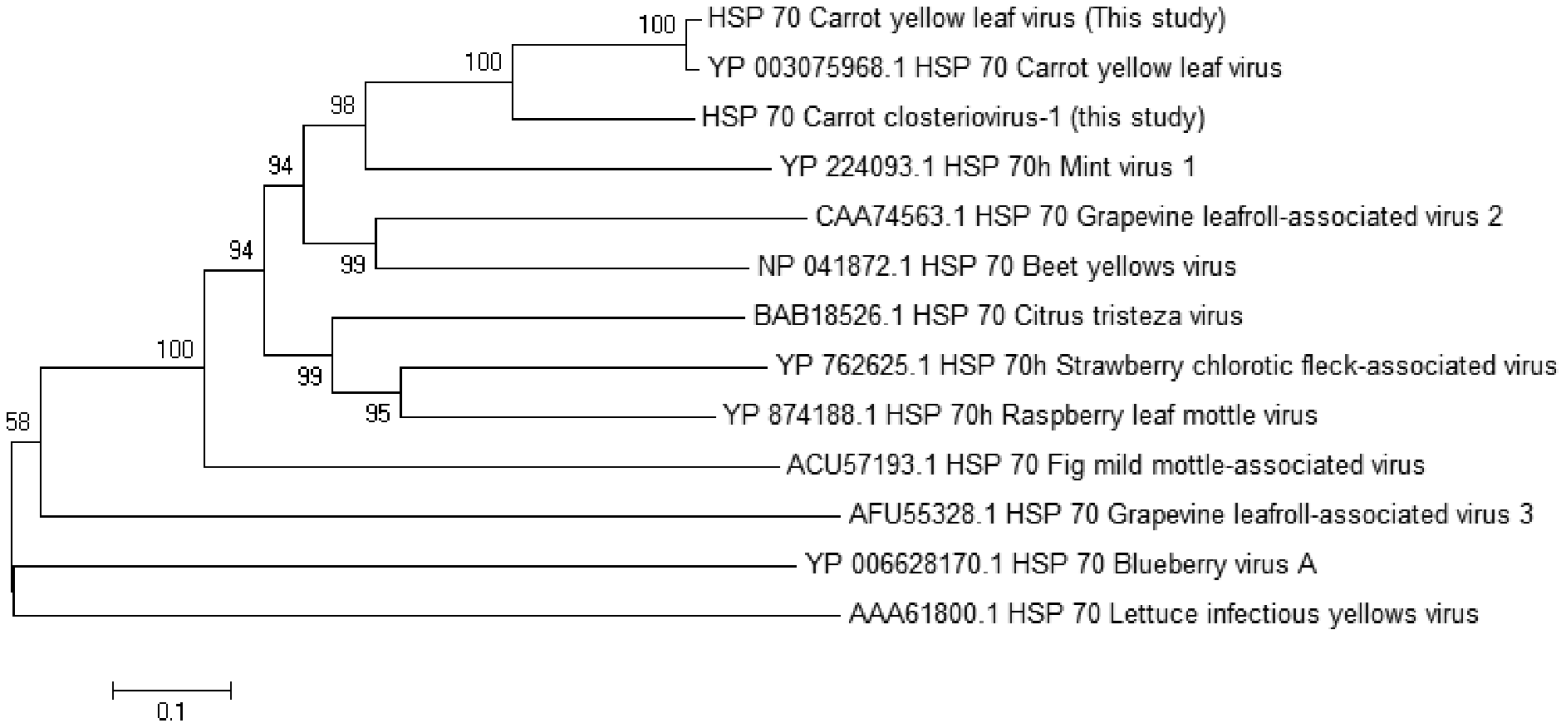
Bootstrapped neighbour joining tree of HSP70 proteins from viruses within the family *Closteroviridae* constructed with MEGA5 using 500 replicates.

Full genomes for CYLV were assembled from reads obtained from all but one of the affected samples, while only small (<1000 bp) fragments were assembled from reads obtained from the unaffected samples. The genome sequences (KF533698–KF533708) were conserved between samples (>98% nucleotide identity) and closely related (95% nucleotide identity) to the reference genome for CYLV [31] (Acc NC013007.1). CYLV was not detected in one of the affected samples, but this sample was found to be infected with a novel virus related to CYLV. This virus, tentatively named Carrot closterovirus 1 (CtCV-1) for the purposes of this study, has a genome of 19923 nucleotides (KF533697). Open reading frame analysis identified 9 open reading frames analogous to 9 of the open reading frames found in CYLV. CtCV-1 lacks ORF 4 found in CYLV but a related virus *Mint virus 1* also lacks this open reading frame. Examination of the amino acid sequences of the putative coat, polymerase and HSP70h proteins suggest that CtCV-1 is a distinct member of the genus *Closterovirus*. When compared to its closest sequenced relative CYLV, the polymerase of CtCV-1 is 84% homologous, the HSP70h 64% homologous and the two coat proteins 69% and 46% homologous respectively. The species demarcation for closteroviruses specifies less than 75% homology in these values [32]. The coat proteins of CtCV-1 (24.7 kDa, 23.3 kDa) are also slightly larger than those of CYLV (24.5 kDa, 22.7 kDa). Figure 4 shows a phylogenetic tree constructed using the HSP70h sequences of CtCV-1, CYLV and related closteroviruses, again confirming CtCV-1 as a distinct member of the genus *Closterovirus*.

Almost complete genomes of CtRLV were recovered from 3 of the unaffected samples (KF533716–KF533718) but not from the affected samples. Small fragments of CtRLVaRNA were recovered from one of the unaffected samples (KF533715) which was also infected with CtRLV. A large fragment (2 kb, KF533709) was recovered from one unaffected sample, which was also infected with CtRLV. The analysis of this fragment showed 94% sequence identity to Beet western yellows virus associated RNA (BWYVaRNA) and correspond to 75% of the complete genome.

Complete genomes of CMoV were recovered from 1 affected and 2 unaffected samples (KF533712–KF533714). These sequences have between 91–96% identity to the complete genome of CMoV (Acc: FJ88473).

**Figure 5:**
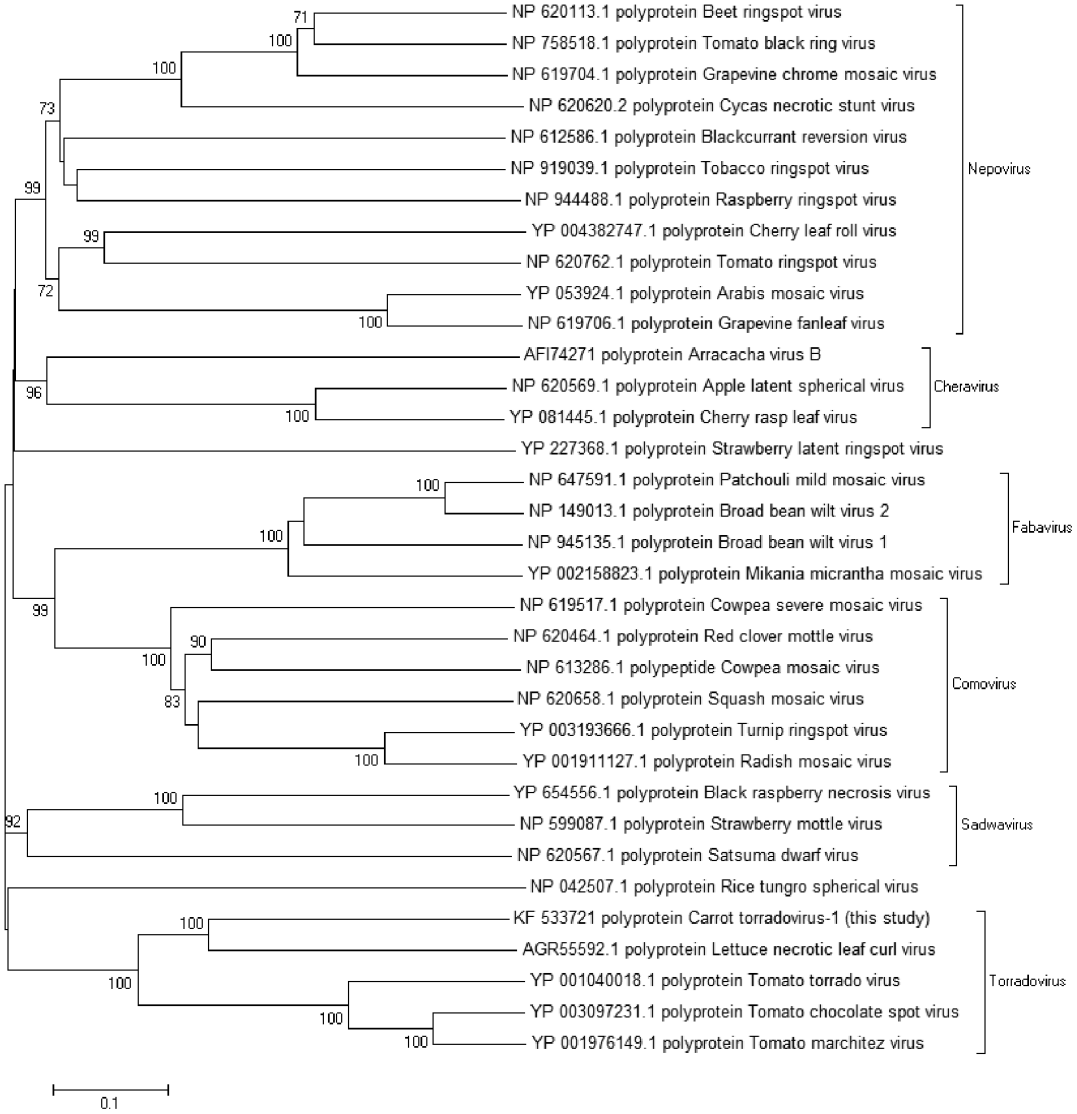
Bootstrapped neighbour joining tree of RNA2 polyproteins from viruses within the family *Secoviridae* constructed with MEGA5 using 500 replicates.

Over 10,000 reads of a novel unclassified ssRNA positive strand virus were found in the unaffected and affected samples. Large fragments (6.9 k and 4.7 k nucleotides) of a bipartite viral genome were found in one affected and one unaffected sample and smaller fragments in another unaffected sample. The fragments from the two different samples were>99% identical suggesting that they were infected with the same virus. Analysis suggests that the 6.9 k nucleotide fragment is the RNA1 genome of a novel *Torradovirus* tentatively named Carrot torradovirus 1 (CTV-1) (KF533719). It contains an open reading frame coding for a 2214 amino acid (249 kDa) polypeptide. BLAST analysis of this putative protein showed it to contain RNA helicase and RdRp domains and have 40% homology to the equivalent protein sequences from *Tomato marchitez virus* [33] and Tomato chocolate spot virus [34]. The 4.7 k nucleotide fragment appears to be the RNA2 genome of a novel torradovirus (KF533720). This contains two open reading frames ORF1 encoding a putative 202 amino acid (22 kDa) protein with 43% homology to the RNA2 ORF1 from Lettuce necrotic leaf curl virus, a recently reported torradovirus (KC855266). This rate of homology within ORF1 leads to a clear demarcation with the genus *Torradovirus*. The second ORF encodes a putative 1167 amino acid (130 kDa) polyprotein. This polyprotein appears to contain movement and coat protein domains and have 35% homology to the RNA2 ORF2 from *Tomato torrado virus*. A phylogenetic tree (figure 5) constructed using the RNA2 polyprotein sequences from torradoviruses and other viruses from the *Secoviridiae* shows the closest related torradovirus to be Lettuce necrotic leaf curl virus.

**Figure 6:**
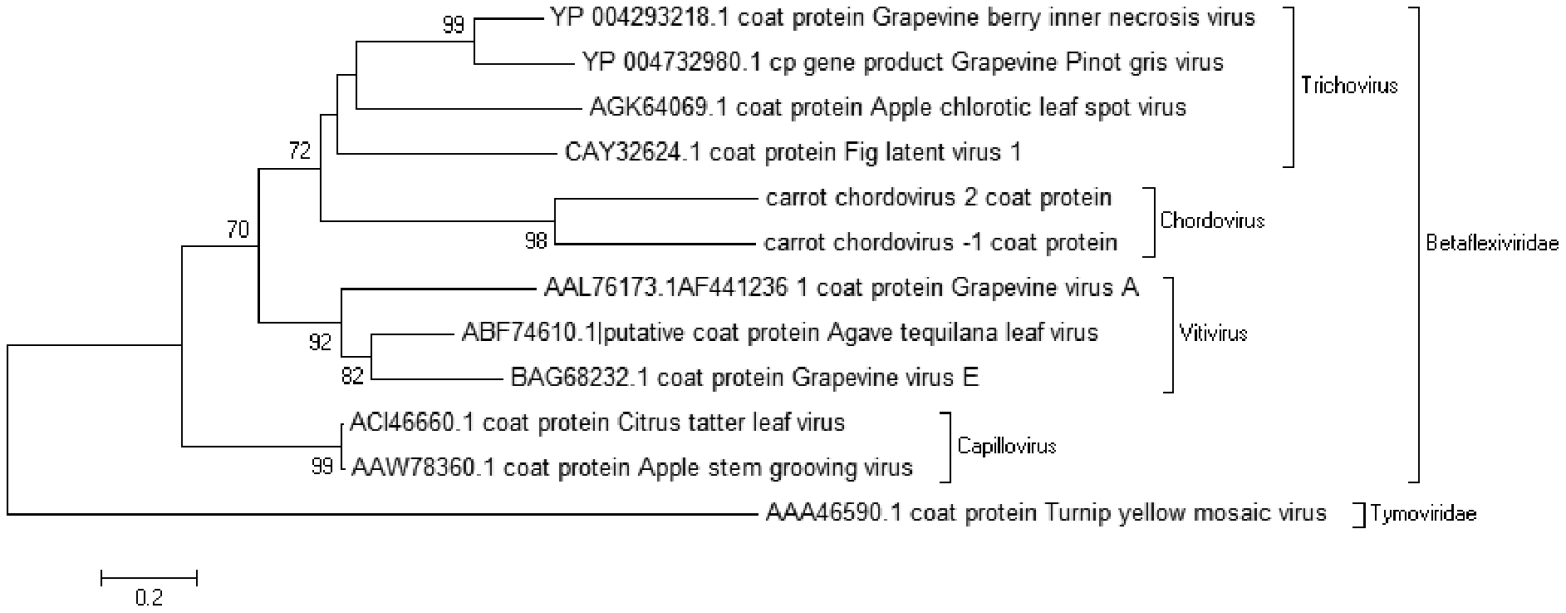
Bootstrapped neighbour joining tree of coat proteins from viruses within the family *Betaflexiviridae* constructed with MEGA5 using 500 replicates.

Over 1000 reads with identity to the family *Betaflexiviridae* were found mainly in the affected samples. Table 1 details the relative abundance (expressed as RPKM) of the identified viruses in the affected and unaffected samples. Further examination revealed that 5 affected samples contained large fragments (>5 k nucleotides) and 3 other affected and 1 unaffected samples had fragments between 1000–5000 nucleotides. Analysis of the 5 larger fragments suggests that they are derived from 2 distinct viruses 1 from 4 samples tentatively named Carrot chordovirus-1 (CtChV-1) (KF533711), 1 from the fifth sample, tentatively named Carrot chordovirus-2 (CtChV-2) (KF533710). Both viruses have genomes of approximately 8.5 k nucleotides and encode 3 putative proteins expected in the *Betaflexiviridae*. The putative coat protein and polymerase nucleotide sequences have less than 45% identity to any previously sequenced virus suggesting they may constitute a new genus [32], within the *Betaflexiviridae*, tentatively named Chordovirus. Comparison of the putative coat protein and polymerase nucleotide and amino acid sequences suggests that the two viruses are in the same genus (>45% nucleotide identity) but distinct viruses 40% amino acid homology within the coat protein. Figure 6 shows a phylogenetic tree produced using the coat protein sequences of related members of the family *Betaflexiviridae* and further providing evidence that these viruses may constitute a novel genus.

**Table 1:** Relative abundance (Reads per kilobase of viral genome per million sequenced reads-RPKM) of select viruses in sequenced affected and unaffected samples.

virus	unaffected	affected
CYLV	2.2	221.3
CtCV-1	0.0	22.0
CTV-1 RNA1	36.4	3.6
CTV-1 RNA2	15.9	5.1
CtRLV	16.2	1.4
CtChV-1	0.0	2.3
CtChV-2	0.0	2.1
CMoV	4.0	4.2

### Sequencing follow-up

Following the outcomes of sequencing, real-time RT-PCR (TaqMan) assays were designed to CYLV and CtCV-1 as follows: CYLV Forward: 5′-AAGATTCTCTTGTAACGAAGGTTTCC, reverse: 5′-GCCGCCTCCACGATCAC, Probe: 5′ Fam-AGACCTCACTATGCTAAACCCGAGCCGG-Tamra. CtCV-1 Forward: 5′-GCCTCCCGCTTGTTGGA reverse: 5′-AGCCGCCAACGTCTATGAAG Probe 5′ Fam-AATAGGACCGTCGCGAGTTTCTGCTCTG-Tamra.

These assays were then used to test the nucleic acid extracts of the 24 carrot sub-sample which had been analysed by sequencing. Of these only 1 of the affected carrots contained CtCV-1, with all 12 affected carrots testing positive for CYLV. Three of the unaffected carrot roots tested positive for CYLV, though in two of these cases the virus was detected at weak levels (>39 Ct).

On the basis of this finding the nucleic acid extracts from the field samples (affected and unaffected carrots) which had been previously tested using RT-PCR were tested for the presence of CYLV using the real-time RT-PCR assay. The results (Figure 2) show that of the carrots affected by necrotic symptoms 98% (99 of 102 carrots tested) were found to be positive, whilst in the unaffected sub-sample 22% (22 of 99 carrots tested) contained the virus. It was also found that the 3 affected samples which tested negative for CYLV tested positive for CtCV-1.

Attempts were made to sap inoculate CYLV from infected carrots into healthy carrots and other species of indicator plants. No infection was detected.

### Statistical analysis

The crop of carrots sampled showing 3% of necrotic symptoms i.e. 3300 carrots were sliced to obtain 100 symptomatic samples. Some of these carrots had surface necrosis which could have been graded out, others had only internal necrosis, and there were also carrots sampled with a combination of internal and external symptoms with approximately even numbers of each within the sample set.

The statistical analysis is presented in Table 2. The results for carrot roots found to be positive for the established carrot viruses (PYFV, CtRLV, CtRLaVRNA and CMoV) show a similar proportion of carrots with and without necrosis indicating that necrosis is probably independent of infection with these viruses. With one exception (*Carrot yellow leaf virus*, CYLV) there is no association between virus status and the prevalence of necrosis. CYLV-positive carrots have an estimated prevalence of necrosis of 12.0% (8.4–17.1%) while the prevalence of necrosis in CYLV-negative carrots is estimated to be 0.1% (0.0–0.3%). The estimated reduction in necrosis prevalence associated with the removal of a virus can be seen in table 2 (Column E%). Removing CYLV from the population is estimated to have a large potential effect, with an estimated reduction of 96% (89.6–98.8%). of necrosis. Because necrosis without CYLV, even in the presence of other viruses, is estimated to be rare (0.0–0.3%) the removal of CYLV alone may be sufficient to greatly reduce the prevalence of necrosis if CYLV is indeed causative.

**Table 2:** Estimates of prevalence of virus and necrosis and the effect of virus removal on reducing necrosis (values in brackets are 95% confidence intervals).

Virus	P(V|N) (%)	P(V|∼N) (%)	P(V) (%)	P(N|V) (%)	P(N|∼V) (%)	E (%)
**PYFV**	2.0 (0.4–6.1)	6.1 (2.6–12.1)	5.9 (2.5–12.0)	1.0 (0.1–4.2)	3.1 (2.5–3.8)	−4.2 (−10.8–1.3)
**CtRLV**	32.4 (23.9–41.8)	27.3 (19.2–36.6)	27.4 (19.7–36.3)	3.5 (2.2–5.5)	2.8 (2.1–3.6)	6.8 (−11.3–21.8)
**CtRLVaRNA**	0.0 (0.0–2.9)	0.0 (0.0–3.0)	0.0 (0.0–3.5)	NE	NE	NE
**CMoV**	8.8 (4.5–15.5)	14.1(8.3–22.0)	14 (8.3–21.9)	1.9 (0.8–4.2)	3.2 (2.5–3.9)	−6.0 (−17–3.7)
**CYLV**	97.1 (92.4–99.2)	22.2 (14.9–31.1)	24.5 (17.1–33.1)	12.0 (8.4–17.1)	0.1 (0.0–0.3)	96.1 (89.6–98.8)
**Any positive**	98 (93.9–99.6)	52.5 (42.7–62.2)	53.9 (44.3–63.5)	5.5 (4.2–7.1)	0.1 (0.0–0.4)	95.7 (86.5–99.1)
**CtRLV+CMoV**	0.0 (0.0–2.9)	4.0 (1.4–9.3)	3.9 (1.4–9.1)	0.0 (0–17)	3.1 (2.5–3.7)	−4.1 (−9.2–0.4)
**PYFV+CMoV**	0.0 (0.0–2.9)	1.0 (0.1–4.6)	1.0 (0.1–4.6)	0.0 (0–17)	3.0 (2.4–3.6)	−1.0 (−4.2–2.5)
**PYFV+CtRLV**	0.0 (0.0–2.9)	1.0 (0.1–4.6)	1.0 (0.1–4.6)	0.0 (0–17)	3.0 (2.4–3.6)	−1.0 (−4.1–2.6)
**PYFV+CYLV**	1.0 (0.1–4.5)	0.0 (0.0–3.0)	0.0 (0–3.7)	100 (0.4–100)	3.0 (2.4–3.6)	1.0 (−2.5–3.7)
**CtRLV+CYLV**	26.5 (18.7–35.6)	7.1 (3.2–13.4)	7.7 (3.9–13.7)	10.4 (5.2–21.7)	2.4 (1.9–3.0)	20.4 (10.2–30.4)
**CMoV+CYLV**	2.9 (0.8–7.6)	0.0 (0.0–3.0)	0.1 (0.1–3.5)	100 (2.2–100)	2.9 (2.4–3.5)	2.9 (−1.3–6.5)
**PYFV, CtRLV +CMoV**	0.0 (0.0–2.9)	1.0 (0.1–4.6)	1.0 (0.1–4.5)	0.0 (0.0–24.3)	3.0 (2.4–3.6)	−1.0 (−4.2–2.3)
**CtRLV, CMoV +CYLV**	4.9 (1.9–10.4)	1.0 (0.1–4.6)	1.1 (0.3–4.5)	13.1 (2.4–61)	2.9 (2.3–3.5)	3.8 (−0.8–9.1)
**PYFV, CMoV +CYLV**	1.0 (0.1–4.5)	0.0 (0.0–3.0)	0.0 (0.0–3.6)	100 (0.4–100)	3.0 (2.4–3.6)	1.0 (−2.8–3.8)

Virus is presented both singly and in combinations. **P(V|N)**: proportion of necrotic carrots with the virus, **P(V|∼N)**: proportion of non-necrotic carrots with the virus, **P(V)** prevalence of virus across all carrots, **P(N|V)**: proportion of carrots with the virus that are necrotic, **P(N|∼V)**: proportion of carrots without the virus that are necrotic. **E**: estimated effect of removing the virus on the prevalence of necrosis expressed as a proportional reduction in the prevalence of necrosis, **NE**: not estimated.

## Discussion

Carrot roots exhibiting internal necrosis have been a growing problem in the UK. Due to a lack of methods allowing the rapid screening of both symptomatic and asymptomatic roots for the presence of a broad range of pathogens, progress on identifying the causal pathogen has been limited.

Of the thirty or so viruses known to have carrot as a host, at least twelve are known to be present in the UK. However, these viruses are not amongst those regularly tested for by diagnostic labs either due to unknown prevalence, poor symptomatic recognition, or more commonly, poor availability of targeted diagnostics. As a result this study has applied high throughput sequencing to screen carrots to help identify a putative causative agent for internal necrosis and a range of previously un-described viruses.

Testing for the four most common viruses (PYFV, CMoV, CtRLV, CtRLVaRNA) in affected and unaffected carrot samples did not provide any evidence for a link between any single or group of viruses and necrosis.

The data obtained following high-throughput sequencing showed that *Carrot yellow leaf virus* was present in eleven of the affected samples and was by far the most common virus recovered warranting further study. The prevalence of this virus was much lower in the unaffected samples. The statistical analysis clearly indicates a link between this unexpected virus finding and the presence of necrosis in carrot roots at this site. Indeed if CYLV is the causal pathogen of carrot internal necrosis, removing CYLV from the sampled carrot population would give an estimated effect of reducing the incidence of necrosis by 96%. Demonstrating a mathematical statistical relationship does not show that there is biological causative relationship but it does point towards where further investigations should be carried out.

Attempts were made to sap inoculate CYLV into carrot or other indicator plants to carry out Koch's postulates but this proved unsuccessful. Previously Murant [8] reported that sap inoculation had been unsuccessful whereas van Dijk and Bos [9] reported poor sap transmissibility into *Nicotiana benthamiana*. Discussion with the authors of the 2009 paper on CLYV [31] also confirmed that they had been unable to sap inoculate CYLV. CYLV is aphid transmitted. In order to transmit CYLV using captive aphids live carrots with attached leaves would be required. In the current study the necrotic symptoms were determined by cutting the carrot root on the grading line when the carrot had already been harvested and the leaves removed. To date it has not been possible to obtain a live symptomatic carrot plant, therefore it has not been possible to further characterize the effects of CYLV on carrot root necrosis.

Due to the low incidence of expression (3%) and localised nature of these symptoms, it was decided that the strategy most likely to yield informative results was to focus in depth on an affected crop from a single site, sampled at the point where symptoms were evident (i.e. on the processing line). It is appreciated that caution should be applied in extrapolating from a single sampled site to other affected sites and future work will include a multi-site survey to confirm the applicability of these findings in a broader context. The statistical approach used in this study, based upon testing approximately equal numbers of affected and unaffected individuals for the presence of pathogens can be applied where infection and disease are often anecdotally linked but lack an empirically observed basis. This could be of particular use when applied to diseases thought to be caused by obligate pathogens such as viruses, viroids, phytoplasmas, or fungal obligates such as rusts or powdery and downy mildews.

Sequencing also identified sequences from 3 of the known carrot infecting viruses CtRLV, CtRLVaRNA and CMoV. CMoV was detected in both affected and unaffected samples by both methods. CtRLV was found by PCR and sequencing to be present in both affected and unaffected samples. CtRLVaRNA was not detected by the conventional PCR [21] but small fragments were detected by sequencing. This might be due to specificity issues with the PCR assay. Therefore the new sequence may prove useful for improving the currently used primer sets. Conversely PYFV was detected by conventional PCR in 2% of affected and 6% of unaffected samples but none was found by sequencing, perhaps indicating the PCR approach is more sensitive than sequencing.

BWYVaRNA sequences were recovered from a sample also containing CtRLV. As far as we are aware, this is the first example of an association between these two viruses and deserves further examination as to whether BWYVaRNA is encapsidated by the CtRLV coat protein.

Four new viruses were identified in the sequencing data. A new closterovirus tentatively named CtCV-1, closely related to CYLV was found in necrotic carrots in the absence of CYLV. This suggests that CtCV-1 may also have a role in carrot necrosis and is worthy of further study. A novel torradovirus CTV-1 was found in affected and at a higher abundance in unaffected carrots whilst two novel betaflexiviruses CtChV-1 and CtChV-2 were found at low abundance predominantly in affected carrots. These viruses do not appear to be correlated to the necrotic root symptoms central to this study, but may be worthy of further investigation as they may be associated with other carrot diseases or be causing a reduction of crop yield. The novel viruses found within this small study demonstrate the limited knowledge of viral populations in carrots and it can be speculated in other crops also. The impact these viruses are having on crops are certainly not clear, however, these studies provide some of the knowledge (sequence data) and tools (specific tests) to enable us to investigate these effects in the future.

The overall aim of the project was to investigate viral causes of carrot root necrosis. The outcomes of this work show that, although potentially damaging to carrots in terms of lowering yield and causing growth defects, infection by the four established carrot viruses did not correlate with internal necrosis. There is a clear statistical association between the presence of internal necrosis and infection with CYLV. A closely related yet distinct virus tentatively named Carrot closterovirus-1 (CtCV-1) may yet be associated with necrosis, all be it with a lower incidence. This is the first report of a root necrosis symptom in carrot being associated with *Carrot yellow leaf virus*. On the basis of these findings, work is ongoing to demonstrate a biological causal relationship (Koch's Postulates) between CYLV and root necrosis.
